# Integrated mRNA and Small RNA Sequencing Reveals a microRNA Regulatory Network Associated with Starch Biosynthesis in Lotus (*Nelumbo nucifera* Gaertn.) Rhizomes

**DOI:** 10.3390/ijms23147605

**Published:** 2022-07-09

**Authors:** Yamei Zhu, Shuping Zhao, Kangming Deng, Peng Wu, Kai Feng, Liangjun Li

**Affiliations:** 1 School of Horticulture and Plant Protection, Yangzhou University, Wenhui East Road No. 48, Yangzhou 225000, China; zhuyamei001@163.com (Y.Z.); zhaoshuping@yzu.edu.cn (S.Z.); h844198419@163.com (K.D.); wupeng@yzu.edu.cn (P.W.); fengkai@yzu.edu.cn (K.F.); 2 Joint International Research Laboratory of Agriculture and Agri-Product Safety of Ministry of Education of China, Yangzhou University, Yangzhou 225000, China

**Keywords:** *Nelumbo nucifera* Gaertn., starch, RNA-seq, sRNA-seq, miRNA

## Abstract

Internode starch biosynthesis is one of the most important traits in lotus rhizome because of its relation to crop productivity. Understanding the microRNA (miRNA) and mRNA expression profiles related to lotus internode starch biosynthesis would help develop molecular improvement strategies, but they are not yet well-investigated. To identify genes and miRNAs involved in internode starch biosynthesis, the cDNA and small RNA libraries of Z6-1, Z6-2, and Z6-3 were sequenced, and their expression were further studied. Through combined analyses of transcriptome data and small RNA sequencing data, a complex co-expression regulatory network was constructed, in which 20 miRNAs could modulate starch biosynthesis in different internodes by tuning the expression of 10 target genes. QRT-PCR analysis, transient co-expression experiment and dual luciferase assay comprehensively confirmed that *Nnu*miR396a down-regulated the expression of *NnSS2* and ultimately prevents the synthesis of amylopectin, and *Nnu*miR396b down-regulated the expression of *NnPGM2* and ultimately prevents the synthesis of total starch. Our results suggest that miRNAs play a critical role in starch biosynthesis in lotus rhizome, and that miRNA-mediated networks could modulate starch biosynthesis in this tissue. These results have provided important insights into the molecular mechanism of starch biosynthesis in developing lotus rhizome.

## 1. Introduction

*Nelumbo nucifera* Gaertn. is a perennial aquatic herb of Nelumbonaceae, native to China and India. It is a characteristic aquatic vegetable with the largest cultivation area in China [1,2]. According to the statistics of the national characteristic vegetable industry technology system, the national planting area of lotus root is 600,000 hm^2^ in 2020. Fresh lotus rhizome and its processed products are not only resistant to storage and transportation, but also rich in nutrients such as starch, protein, vitamins, flavonoid, and mineral substances, and is famous for its nutritional and medicinal values, which are deeply loved by consumers at home and abroad [3,4]. According to Compendium of Materia written by Shizhen Li, “The lotus decocted with water could detoxify fungus poison and prevent blood collapse and blood drowning.” [5]. The starch content accounting for more than 70% of the total dry rhizome matter is the main factor affecting lotus rhizome edible quality [6].

Starch is a complex branched polymer composed of amylose and amylopectin [7]. The biosynthesis of starch begins in leaves and then transported to other storage organs in the form of disaccharides for starch synthesis. Sucrose transported from leaves to storage organs is successively catalyzed by sucrose synthase (SUS), UDP glucose pyrophosphorylase (UDPase) and phosphoglucomutase (PGM) to produce glucose-6-phosphate, which is then transported into amyloplast for starch synthesis, which is catalyzed by PGM, ADP-glucose pyrophosphorylase (AGPase), starch synthase (SS) starch branching enzyme (SBE) and starch debranching enzyme (DBE) [7,8,9]. Thus far, many genes associated with rhizome development have been researched in lotus. Among them, *SUS* [10], *UDP* [11], *PGM* [12,13], *AGP* [14] are closely related to the synthesis of total starch, *GBSS* [15,16] is a key gene affecting amylose synthesis; *SS* [17,18], *SBE* [17] and *DBE* [19,20] are involved in the synthesis of amylopectin.

At present, miRNAs have been reported to participate in the regulation of starch biosynthesis. MicroRNAs (miRNAs), which constitute a major class of endogenous small RNAs in plants, affect a multitude of developmental and physiological processes by imparting sequence specificity to gene regulation. miRNAs have emerged as important regulators and promising tools for horticultural crop improvement [21]. In cassava, hundreds of miRNAs were covered to potentially be involved in starch metabolism and development in storage roots through sRNA sequencing. Among then, miR394 can target sugar metabolism-related genes to regulate starch biosynthesis [22]. During rice grain filling, a novel miRNA, miRn45-5p, was validated to target a putative sucrose-phosphate synthase (SPS) thus involved in starch synthesis [23]. Two miRNAs, namely miR1436 and miR1439, both cleaving mRNAs encoding starch synthase (SS), have been identified by scanning degradome data and 5′ Race [24]. In potato, miR156, miR529, miR112 and miR1533 might be responsible for starch and sucrose metabolism [25].

Previous studies on the rhizome of lotus root mainly focused on different varieties [26] and different development stages [27,28], but without study on rhizomes at different internodes. Through the combination of high-throughput sequencing technology and bioinformatics, this study intends to explore the influence of miRNA and its target genes on starch synthesis of lotus rhizome at different internodes, verify their functions, and reveal the mechanism of lotus internodes starch synthesis mediated by miRNA, as well as the corresponding target genes and their regulatory relationship.

## 2. Results

### 2.1. Starch Content and Starch Granule Morphology of Lotus Rhizome

**Figure 1:**
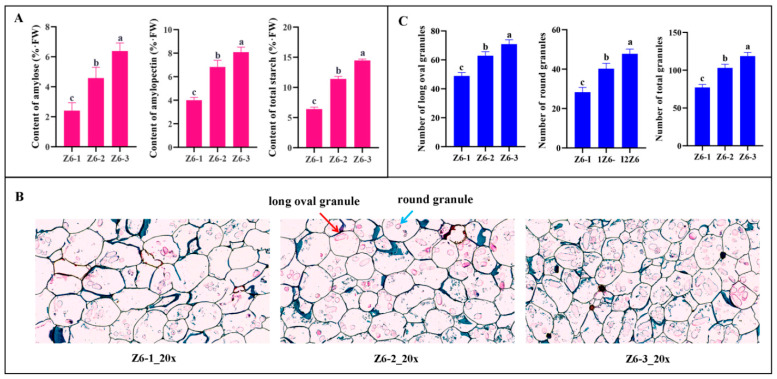
Starch characteristics of lotus rhizome. (**A**) Content of amylose, amylopectin, and starch of Z6-1, Z6-2 and Z6-3. (**B**) The starch granules in paraffin internodes were observed under microscope of Z6-1, Z6-2 and Z6-3. The red arrow represents long oval granule, blue arrow represents round granule. (**C**) Statistics of starch granules of Z6-1, Z6-2 and Z6-3. Each sample used to determinate of amylose, amylopectin and total starch were set for three biological replicates. Ten paraffin section pictures were used to counted the number of starch granules. Error bars show SD from three biological replicates. The ‘a’, ‘b’ and ‘c’ above the histogram indicated the statistical significance at the level of 0.05 (*p* < 0.05).

The content of amylose, amylopectin, and starch had significant difference in three internodes of lotus rhizome, which had upward trend from Z6-1 to Z6-3 (Figure 1A). In paraffin section observation, there existed two types of granules, long oval starch granules and round starch granules, which may indicate the ratio of amylose and amylopectin [5]. The results of statistics of two forms of starch granules showed that the number of long oval starch granules, round starch granules and total starch granules were highest in Z6-1, second in Z6-2 and lowest in Z6-3 (Figure 1B,C), which were consistent with those of starch determination. The difference of starch synthesis was due to Z6-3 growing earliest, where in the later stage of starch synthesis, starch synthesis was basically completed. While Z6-1 was beginning to develop, it stayed in the vigorous period of starch synthesis.

### 2.2. Transcriptome Analysis of Different Internodes in Lotus

**Figure 2:**
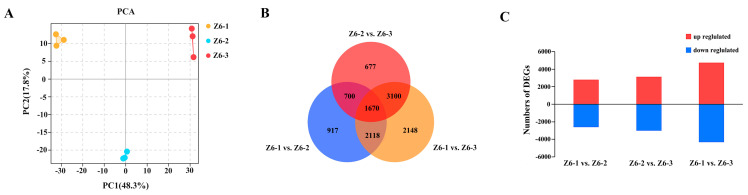
RNA sequencing analysis of different internodes of lotus. (**A**) PCA analysis of Z6-1, Z6-2, and Z6-3. (**B**) Venn diagrams of differentially expressed genes (|log2FC| ≥ 1 and FWER ≤ 0.05) on pairwise comparisons. (**C**) Numbers of up- and down-regulated genes between Z6-1 vs. Z6-2, Z6-2 vs. Z6-3 and Z6-1 vs. Z6-3.

Significant differences in amylose, amylopectin, and total starch contents of three internodes in lotus were observed. To further investigate the regulation of genes related to starch synthesis of different internodes, RNA-seq was performed on the different internode samples. All replicates of each condition clustered together in a principal component analysis (PCA) on log-transformed counts, demonstrating that no obvious sample outliers are present in the dataset. The distinct position of the Z6-1, Z6-2, and Z6-3 indicating significant changes among the internodes in the transcriptome (Figure 2A). The Venn diagram showed that 1670 differentially expressed genes overlapped among the three comparison groups. Most transcriptional changes were observed between Z6-1 and Z6-3 internodes, with >9000 genes being specifically regulated differently at these two internodes. This fitted the great difference of starch between Z6-1 and Z6-3 (Figure 2B). Between the Z6-1 and Z6-2 internodes, 2813 genes were upregulated and 2592 genes were down-regulated. 3129 and 3018 genes increased and decreased at Z6-1 internode, compared with Z6-2 internode. The most differentially expressed genes were in the comparison between Z6-1 and Z6-3, which included 4734 upregulated and 4302 down-regulated genes (Figure 2C).

**Figure 3:**
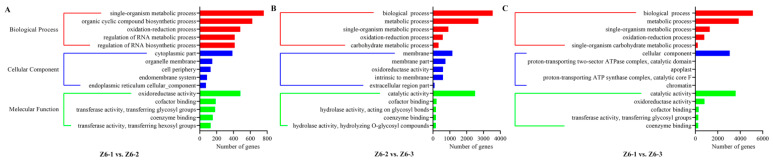
GO enrichment analysis of DEGs on pairwise comparisons. (**A**) Comparison of Z6-1 vs. Z6-2. (**B**) Comparison of Z6-2 vs. Z6-3. (**C**) Comparison of Z6-1 vs. Z6-3.

Generally, gene ontology (GO) was used to classify the functions of the assembled transcripts. The results were distributed into three classes such as biological process, cellular component, and molecular function. Most transcripts were enriched in biological processes, mainly contained “single-organism metabolic process” on Z6-1 vs. Z6-2, and “biological process” both on Z6-2 vs. Z6-3 and Z6-1 vs. Z6-3 (Figure 3).

**Figure 4:**
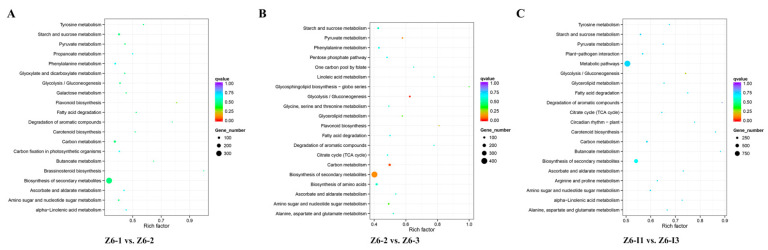
KEGG pathway enrichment analysis of DEGs on pairwise comparisons. (**A**) Comparison of Z6-1 vs. Z6-2. (**B**) Comparison of Z6-2 vs. Z6-3. (**C**) Comparison of Z6-1 vs. Z6-3.

To better understand the function and gene regulatory network, KEGG analysis was carried out. Interestingly, KEGG pathway analysis showed that a large number of DEGs were enriched in “Sucrose and starch metabolism”, “Carbon metabolism”, “Biosynthesis of secondary metabolites” during comparison groups on Z6-1 vs. Z6-2, Z6-2 vs. Z6-3, and Z6-1 vs. Z6-3. Starch synthesis was belong to “Biosynthesis of secondary metabolites” and had inextricably linked with “Carbon metabolism”. Combined with the results of starch content, “Sucrose and starch metabolism” may be the most key pathway deserved our attention (Figure 4).

### 2.3. Expression Profile of Starch Biosynthetic Genes in Different Internodes

**Figure 5:**
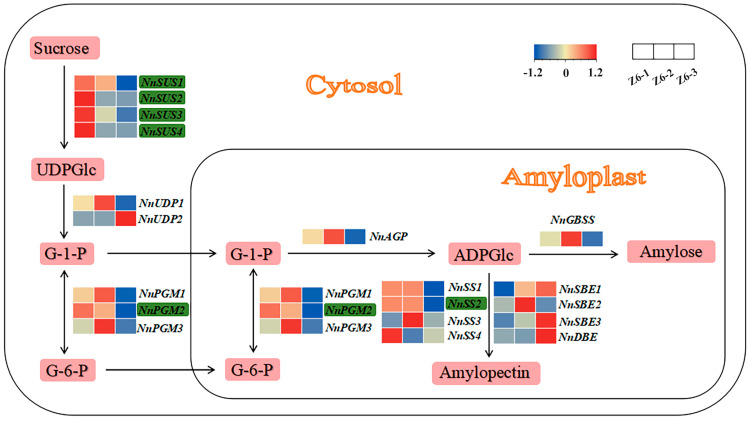
Expression profiles of starch biosynthetic genes. Green rectangles highlight genes down regulated from Z6-1 to Z6-3.

RNA-seq analysis showed that 75 known genes enriched in functional categories “Sucrose and starch metabolism”. When Z6-1 compared with Z6-2, 80 known genes enriched in “Sucrose and starch metabolism”. Overall, 104 known genes enriched in “Sucrose and starch metabolism” when Z6-1 compared with Z6-3. Among them, 19 known genes were related to starch biosynthetic closely. The functions of these 19 genes, and the starch biosynthetic pathways in which they could be involved, are shown in Figure 5. Among these 19 genes, *NnSUS1*, *NnSUS2*, *NnSUS3*, *NnSUS4*, *NnPGM2* and *NnSS2* were down-regulated from Z6-1 to Z6-3, which possibly indicated that these genes are most active in Z6-1 and least expressed on Z6-3. The law of qRT-PCR of the other 13 genes was different from the starch content (Figure 5).

**Figure 6:**
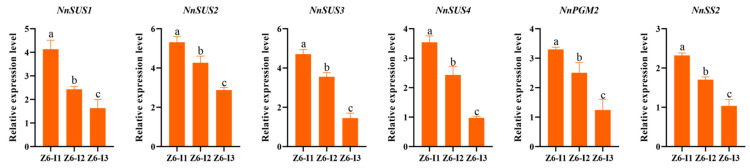
Quantitative RT-PCR validation of selected genes regulated in different internodes. *NnTUA* was used as the reference gene. All the data were calculated with three biological repeats. Error bars show SD from three biological replicates. The ‘a’, ‘b’ and ‘c’ above the histogram indicated the statistical significance at the level of 0.05 (*p* < 0.05).

Quantitative real-time polymerase chain reaction (qRT-PCR) analysis showed that six key starch biosynthetic genes (*NnSUS1*, *NnSUS2*, *NnSUS3*, *NnSUS4*, *NnUDP1*, *NnPGM2*, and *NnSS2*) highly expressed in all three rhizomes. Additionally, the expression of these six genes was the highest in Z6-1, second in Z6-2 and lowest in Z6-3, which is concordantly with the expression profiles obtained from RNA-seq data (Figure 6). The content of amylose, the content of amylopectin, and the content of starch increased from Z6-1 to Z6-3, but the expression of genes was on the contrary. This may be because Z6-1 was in the vigorous period of starch synthesis, while the Z6-3 was basically completed.

### 2.4. Sequencing and miRNAs Identification of Lotus Rhizome in Different Internodes

**Figure 7:**
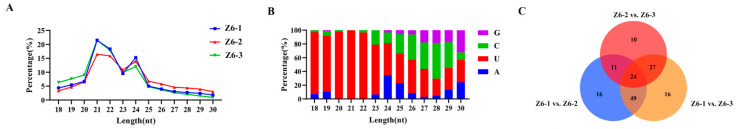
miRNA sequencing analysis of different internodes of lotus. (**A**) Length distribution and percentage analysis of miRNAs from sequencing. (**B**) First nucleotide bias of miRNAs with different lengths. (**C**) Venn diagrams of differentially expressed miRNAs (|log2FC| ≥ 1 and FWER ≤ 0.05) on pairwise comparisons.

Sequencing of small RNAs generated 15,447,040 raw reads containing 15,113,404 unique reads from Z6-1, 14,834,239 raw reads containing 9,596,852 unique reads from Z6-2 and 14,738,812 raw reads containing 14,489,130 unique reads from Z6-3 (Table S1). In total, 480 miRNAs were detected, including 350 known miRNAs and 134 previously unknown ones, and most of which were between 21 and 24 nt in length (Figure 7A). This showed that more miRNAs were obtained from small RNAs sequencing because mature miRNAs are usually 21 or 24 nt in plants due to the specificity of Dicer/DCL [29,30]. Further analysis showed that the first nucleotide of small RNAs getting from sequencing is U, the typical first nucleotide of canonical miRNAs (Figure 7B) [31].

Among all the differently expressed miRNAs, 24 were common in the three comparison groups (Figure 7C), of which 19 were known miRNAs belonging to 5 miRNA families (miR171 family, miR159 family, miR166 family, miR396 family and miR858 family) and 5 were unknown miRNAs.

### 2.5. Targets of Differentially Expressed miRNAs in Different Internodes

**Figure 8:**
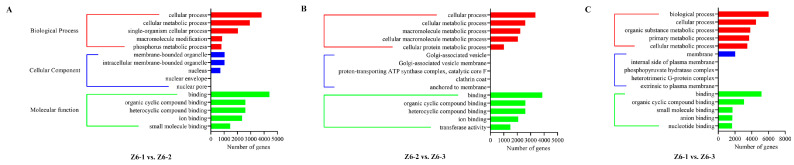
GO enrichment analysis of target genes of differentially expressed miRNAs on pairwise comparisons. (**A**) Comparison of Z6-1 vs. Z6-2. (**B**) Comparison of Z6-2 vs. Z6-3. (**C**) Comparison of Z6-1 vs. Z6-3.

Target genes of these differentially expressed miRNAs were further screened. Based on GO analysis of the targets, it was showed that between Z6-1 and Z6-2, “cellular process” represented the top term in category of biological process, “membrane-bounded organelle” was enriched in cellular component, and the most abundant term was ‘binding’ for the category of molecular function. Between Z6-2 and Z6-3, the targets of differentially expressed miRNAs were significantly associated with “cellular process” in biological processes. Maximal target genes were enriched in cellular components, and “transferase activity” in molecular functions. “Biological process” had most target genes in biological processes, “membrances” were the most abundant term in cellular components, and “binding” represented the top term in category of molecular functions when Z6-I1 compared with Z6-I3 (Figure 8).

**Figure 9:**
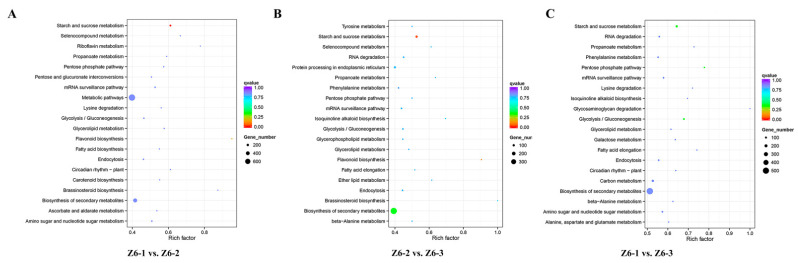
KEGG pathway enrichment analysis of target genes of differentially expressed miRNAs on pairwise comparisons. (**A**) Comparison of Z6-1 vs. Z6-2. (**B**) Comparison of Z6-2 vs. Z6-3. (**C**) Comparison of Z6-1 vs. Z6-3.

KEGG enrichment analysis of the target genes of differentially expressed miRNAs in each comparison group showed that the target genes of differentially expressed miRNAs in the three comparison groups were all significantly enriched in the “Sucrose and starch metabolism” (Figure 9). Among them, most targeted genes of differently expressed miRNAs belonged to the “Sucrose and starch metabolism” pathway in the comparison between Z6-1 and Z6-3 internodes. miRNAs related to starch synthesis in “Sucrose and starch metabolism” pathway and their target genes are screened and displayed in Table S2.

**Figure 10:**
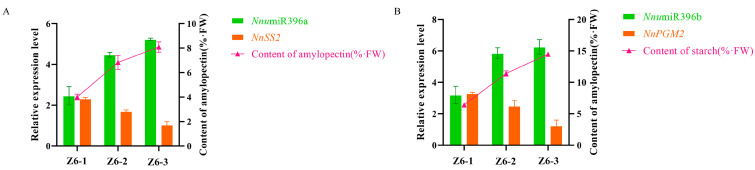
Expression pattern of two key miRNAs and their target genes and starch content in different internodes. (**A**) The expression pattern of *Nnu*miR396a and *NnSS2* and the content of amylopectin. (**B**) The expression pattern of *Nnu*miR396b and *NnPGM2* and the content of starch. *NnTUA* was used as the reference gene. All the data were calculated with three biological repeats. Error bars show SD from three biological replicates.

We analyzed these miRNAs and their target genes by qRT-PCR, combined with the sequencing results, found that *Nnu*miR396a and *Nnu*miR396b had the opposite expression trend with their target genes. We further analyzed the changes of starch content in different internodes to further verify the role of these two miRNAs and their target genes. The results showed that when the amylopectin synthesis rate was fast, the expression of *NnSS2* was relatively high, while the expression of *Nnu*miR396a was relatively low, and the expression of *Nnu*miR396a and *NnSS2* had a negative correlation in three internodes. This indicated that the expression of *NnSS2* advances amylopectin synthesis in different internodes of lotus, while the expression of *Nnu*miR396a was adverse to amylopectin synthesis in different internodes of lotus (Figure 10A). When the total starch synthesis rate was fast, the expression of *NnPGM2* was relatively high, while the expression of *Nnu*miR396b was relatively low, which indicating that the expression of *Nnu*miR396b and *NnPGM2* were negatively correlated in three internodes (Figure 10B).

### 2.6. Validation of the Regulatory Pathways for Starch Biosynthesis Mediated by NnumiR396a-NnSS2 and NnumiR396b-NnPGM2

**Figure 11:**
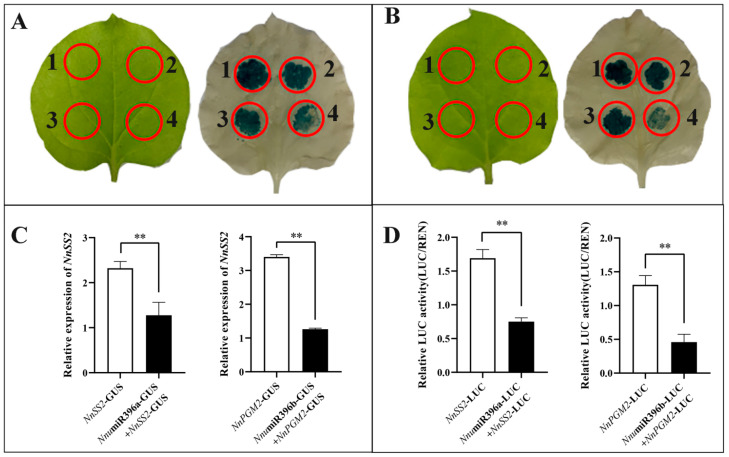
Transient co-expression experiment and dual luciferase assay of two key miRNAs and their target genes. (**A**) Transient co-expression experiment of *Nnu*miR396a and *NnSS2*. 1: pbi121-GUS only, 2: *NnSS2*-GUS only, 3: *Nnu*miRX-GUS + *NnSS2*-GUS, 4: *Nnu*miR396a-GUS + *NnSS2*-GUS. *Nnu*miRX represents one irrelevant miRNA. (**B**) Transient co-expression experiment of *Nnu*miR396b and *NnPGM2*. 1: pbi121-GUS only, 2: *NnPGM2*-GUS only, 3: *Nnu*miRX-GUS + *NnPGM2*-GUS, 4: *Nnu*miR396b-GUS + *NnPGM2*-GUS. *Nnu*miRX represents one irrelevant miRNA. (**C**) Relative expression level of *NnSS2* and *NnPGM2* in tobacco after injection. (**D**) Luc activity of *NnSS2* and *NnPGM2* in tobacco after injection. *NtActin* was used as the reference gene. All the data were calculated with three biological repeats. Error bars show SD from three biological replicates. The ‘**’ above the histogram indicated the statistical significance at the level of 0.01 (*p* < 0.01).

In order to confirm the targeting relationship and regulation mode of *Nnu*miR396a-*NnSS2* and *Nnu*miR396b-*NnPGM2*, we carried out tumefaciens mediated transient co-expression experiment in Nicotiana benthamiana. The results showed that when *Nnu*miR396a-GUS and *NnSS2*-GUS were injected together, the blue fluorescence was clearly shallow than that of *NnSS2*-GUS alone (Figure 11A), and the result of qRT-PCR showed that when *Nnu*miR396a was presented, the expression of *NnSS2* decreased (Figure 11C). When *Nnu*miR396b-GUS and *NnPGM2*-GUS were injected together, the blue fluorescence was clearly shallow than that of *NnPGM2*-GUS alone (Figure 11B), and the result of qRT-PCR showed that when *Nnu*miR396b was presented, the expression of *NnPGM2* decreased (Figure 11C).

To further verify the regulatory relationship between miRNAs and its target genes, we performed a Dual luciferase assay was used to explore the regulatory effect of miRNAs on their target genes. The results show that the relative luciferase level decreased after co-expression of *Nnu*miR396b-LUC and *NnPGM2*-LUC in tobacco leaves than *NnPGM2*-LUC alone, and the relative luciferase level decreased2 after co-expression of *Nnu*-miR396a-LUC and *NnSS2*-LUC in tobacco leaves than *NnSS2*-LUC alone (Figure 11D).

Transient co-expression experiment and dual luciferase assay confirmed that *NnSS2* was the target gene of *Nnu*miR396a, and *NnPGM2* was the target gene of *Nnu*miR396b. Additionally, there was a negative regulatory relationship between the two miRNAs and their target genes.

**Figure 12:**
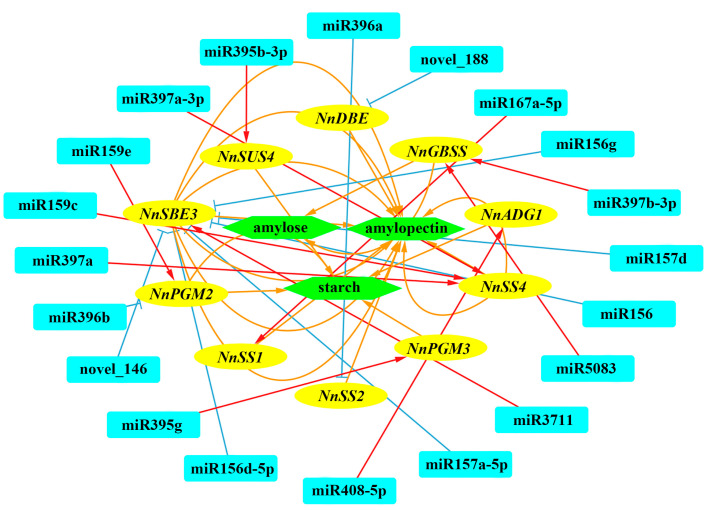
Co-expression network of miRNA-target-starch biosynthesis in different internodes. Blue boxes represent miRNAs, yellow boxes represent target genes, and green boxes represent amylose, amylopectin and total starch.

The miRNA regulates target mRNA through translational repression or mRNA degradation. Co-expression of the correlation between the expression of DEMs and DEGs involved in “Sucrose and starch metabolism” selected from KEGG enrichment were conducted. Notably, 20 miRNAs and 10 genes formed 20 miRNA-target mRNA pairs with co-expressed expression revealed a complex network modulating starch biosynthesis in different internodes (Figure 12). The sequences of related miRNAs are supplemented in Table S3.

## 3. Discussion

Lotus is a characteristic aquatic vegetable with the largest cultivated area in China. The starch content, composition and structure of its rhizome are the main indexes affecting the yield and edible quality of lotus rhizome. In our study, the content of amylose, the content of amylopectin, and the content of starch all had an upward trend from Z6-1 to Z6-3, which showed that the first internode is in the vigorous period of starch development, while the third internode has been preliminarily completed. This is consistent with the rising trend of amylose content, amylopectin content and starch content from the beginning stage to the end stage [5,32].

During the development of lotus, most of the genes related to starch synthesis are expressed actively. [5,32]. *SUS* and *SS* were involved in the changes at development stages of lotus rhizome [26]. *Sucrose synthase (SUS)* is the first key gene in the regulation of sucrose and starch transformation in plants, which converts sucrose into UDP-glucose and fructose [33]. Tuberous roots were shown to possess high *sucrose synthase (SUS)* activity [34]. In our study, *NnSUS1*, *NnSUS2*, *NnSUS3*, and *NnSUS4* were also highly expressed in lotus rhizomes. The transcription of *NnSUSs* were highest in Z6-1, second in Z6-2 and lowest in Z6-3. That presented the expression of *NnSUS1*, *NnSUS2*, *NnSUS3*, and *NnSUS4* were higher in the internodes with faster starch synthesis rate. Phosphoglucomutase (PGM) catalyzes the interconversion of glucose 1-phosphate (G-1-*p*) and glucose 6-phosphate (G-6-P) [35]. *Phosphoglucomutase 2* (*NnPGM2*) gene was significantly down-regulated from Z6-1 to Z6-3, indicating that most glucose-6-phosphate (G-6-P) was converted to glucose-1-phosphate (G-1-P) in Z6-1 [36]. Differentially expressed *starch synthase 2* (*SS2*) genes, code for amylopectin biosynthesis and were down regulated from Z6-1 to Z6-3, showed supreme expression on Z6-1 [37].

As regulation by miRNAs could be a major factor leading to the differential expression of these genes, the regulation by *Nnu*miR396a and *Nnu*miR396b of starch biosynthesis was further analyzed. miR396 is a highly conserved miRNA family and involved in regulating the growth and development of plants by inhibiting the expression of target genes [38]. In rice, miR396c can regulate grain development and yield through the miR396c-OsGRF4 (growth-regulating factor 4)-OsGIF1 (GRF-interacting filling 1) pathway, which might be associated with starch biosynthesis [39]. In wheat, miR396 was involved in the development of grains by regulating the expression of GRF genes [38,40]. MiR396 also participates in the process of maize grain filling by negatively regulating 14 GRF genes, according to computational prediction, RNA sequencing and validated by degradome sequencing [41]. In the present study, *Nnu*miR396a and *Nnu*miR396b down regulated the expression of their target genes to influence the starch synthesis of lotus rhizome.

## 4. Materials and Methods

### 4.1. Plant Materials

Lotus cultivar ‘Z6′ of *N. nucifera* was used in this study, which has typical rhizome enlargement. It was planted on 5 May 2020 in the experimental station of aquatic vegetables, Yangzhou University, JiangSu Province, China. The development of lotus rhizome could be classified into three stages, early swelling (S1), middle swelling (S2), and later swelling stage (S3). Among them, there are three internodes with great development differences in the middle swelling (S2), which is suitable for studying the differences of rhizomes at different internodes. Three lotus roots with similar size were taken at the middle swelling (S2) on 20 August 2020. They were immediately washed, dried, cut into pieces, put in liquid nitrogen and put in refrigerator at −80 °C for reserve. We called the rhizome near the terminal bud the first internode (Z6-1), then the second internode (Z6-2) and the third internode (Z6-3) (Figure S1) [26]. The length and girth of three lotus roots were suppled in Table S4.

### 4.2. Determination of Starch Content

The content of total starch was determined by anthrone colorimetry using the starch content kit (Beijing Solarbio Science & Technology Co., Ltd., Beijing, China). The content of amylose was determined by iodine colorimetry using the amylose content kit (Beijing Solarbio Science & Technology Co., Ltd.). The content of amylopectin = the content of total starch-The content of amylose. Each sample was set for three biological replicates and SPSS software was used to statistical analysis.

### 4.3. Small RNA and Transcriptome Sequencing

In this study, nine libraries of “Z6” (Z6-1-1, Z6-1-2, Z6-1-3, Z6-2-1, Z6-2-2, Z6-2-3, Z6-3-1, Z6-3-2, and Z6-3-3) were constructed for sequencing. sRNA-seq and RNA-seq libraries were constructed and sequenced by Beijing Novo gene Technology Co., Ltd. (Beijing, China). sRNA-seq uses the small RNA sample pre kit to construct the library. Using the special structure of the 3′ and 5′ ends of small RNA (complete phosphate group at the 5′ end and hydroxyl group at the 3′ end), with total RNA as the starting sample, the two ends of small RNA are directly spliced, and then reverse transcribed to synthesize cDNA. After PCR amplification, the target DNA fragment was separated by PAGE gel electrophoresis, and the cDNA library was recovered by cutting gel. RNA-seq extracts total RNA from lotus rhizome samples. After enriching eukaryotic mRNA with Polya tail through magnetic beads with oligo (DT), the mRNA is interrupted by ultrasound. Using fragmented mRNA as template and random oligonucleotides as primers, the first cDNA strand was synthesized in M-MuLV reverse transcriptase system, then RNA strand was degraded by RNase H, and the second cDNA strand was synthesized from dNTPs in DNA polymerase I system. The purified double stranded cDNA was repaired at the end, then added with a tail and connected to the sequencing connector. The cDNA of about 200 bp was screened with ampure XP beads for PCR amplification. The PCR product was purified again with ampure XP beads, and finally the library was obtained.

### 4.4. Bioinformation Analysis of the Data

In this study, nine libraries of Z6-1, Z6-2, and Z6-3 were constructed for sequencing. sRNA-seq and RNA-seq libraries were constructed and sequenced by Beijing Novo gene Technology Co., Ltd. (Beijing, China). sRNA-seq uses the small RNA sample pre kit to construct the library. Using the special structure of the 3′ and 5′ ends of small RNA (complete phosphate group at the 5′ end and hydroxyl group at the 3′ end), with total RNA as the starting sample, the two ends of small RNA are directly spliced, and then reverse transcribed to synthesize cDNA. After PCR amplification, the target DNA fragment was separated by PAGE gel electrophoresis, and the cDNA library was recovered by cutting gel. RNA-seq extracts total RNA from lotus rhizome samples. After enriching eukaryotic mRNA with Polya tail through magnetic beads with oligo (DT), the mRNA is interrupted by ultrasound. Using fragmented mRNA as template and random oligonucleotides as primers, the first cDNA strand was synthesized in M-MuLV reverse transcriptase system, then RNA strand was degraded by RNase H, and the second cDNA strand was synthesized from dNTPs in DNA polymerase I system. The purified double stranded cDNA was repaired at the end, then added with a tail and connected to the sequencing connector. The cDNA of about 200 bp was screened with ampure XP beads for PCR amplification. The PCR product was purified again with ampure XP beads, and finally the library was obtained. The *p*-value of each gene was then corrected by a multiple hypothesis test using Qvalue. A gene was defined as a DEG when reads number fold change ≥2 and Q-value ≤0.01 [42,43]. DEGs were subjected to GO functional enrichment and KEGG pathway analyses. GO annotation was conducted using Blast2GO software (v.2.5.0), and the significance of enrichment of each functional term was computed using the hypergeometric distribution test [44].

### 4.5. QRT-PCR Analysis of mRNAs

RNA isolater total RNA extraction reagent was used to extract total RNA from rhizomes of lotus. Then, HiScriptII RT SuperMixfor qPCR (Vazyme Biotech Co., Ltd., Nanjing, China) was used to reverse transcription into cDNA. The key structural genes of “Sucrose and starch metabolism” pathway in lotus were analyzed by quantitative reverse transcription-polymerase chain reaction (qRT-PCR), used chamQ SYBR qPCR Master Mix (Vazyme Biotech Co., Ltd.). Gene-specific primers were designed according to the reference unigene sequences using Primer 5.0 (Table S5). The *β*-Actin (*NnTUA*, GeneID: 104597659) gene was used as an internal gene expression control: the gene was amplified with forward primer 5′-ACCGCCTCGTCTCTCTTTGG-3′ and reverse primer 5′-CGACCTGAATCCCCGCTTGT-3′. Amplification was performed on the CFX-96 Real-time PCR system (Bio-Rad) using the following real-time fluorescent quantitative PCR program: 95 °C for 30 s, then 95 °C for 10 s and 60 °C for 30 s for a total of 40 cycles. The relative gene expression was calculated by 2^−∆CT^ [45]. Three replicates were performed for each amplification reaction. We used SPSS software for statistical analysis of the qRT-PCR data.

### 4.6. QRT-PCR Analysis of miRNAs

The total RNA was used as qRT-PCR analysis of mRNAs for the qRT-PCR analysis of miRNAs. Then, miRNA 1st starnd cDNA synthesis kit (by stem-loop) (Vazyme Biotech Co., Ltd.) was used to reverse transcription into cDNA. The key miRNAs of “Sucrose and starch metabolism” pathway in lotus were analyzed by quantitative reverse transcription-polymerase chain reaction (qRT-PCR). The qRT-PCR reaction was referenced to miRNA Universal SYBR qPCR Master Mix (Vazyme Biotech Co., Ltd.). The primers of miRNAs were designed according to the reference mature sequences using Vazyme miRNA Design software (Table S6). The U6 gene was used as a control: the stem-loop primer was 5′-TCGATTGTGCGTCATCC-3′ and specific primer 5′-ACAGAGAAGATTAGCATGGCCC-3′, the mQ primer R was provided by Kit. Amplification was performed on the CFX-96 Real-time PCR system (Bio-Rad) using the following real-time fluorescent quantitative PCR program: 95 °C for 1 min, then 95 °C for 10 s and 60 °C for 30 s for a total of 40 cycles. The relative miRNA expression was calculated by 2^−∆CT^ [45]. Three replicates were performed for each amplification reaction. We used SPSS software for statistical analysis of the qRT-PCR data.

### 4.7. Transient Expression Analysis

The pre-*Nnu*miR396a/396b precursor and the coding sequences of *NnSS2* and *NnPGM2* were combined to pbi121-GUS (*Nnu*miR396a-GUS, *Nnu*miR396b-GUS, *NnSS2*-GUS and *NnPGM2*-GUS) (Cat No.10911; Shanghai, China). The recombinant plasmids of miRNA and target genes were transferred into *Agrobacterium* strain GV3101. The bacteria were resuspended with the infection solution (100 mM Acetosyringone, 0.5 M MES (pH5.6) and 10 mM MgCl_2_) until the OD600 value was 1.0, and the bacterial solution containing miRNA and target genes were mixed in a ratio of 1:1, and then left to stand for 3 h. Five-week-old tobacco plants (*Nicotiana benthamiana*) were infiltrated with the suspended bacterical solutions. GUS enzyme activity in tobacco leaves was detected using 5-bromo-4-chloro-3-indolyl-β-D-glucuronide (X-Gluc) as substrate (Beijing Solarbio Science & Technology Co., Ltd.). Total RNA extraction from tobacco leaves and real-time qRT-PCR analysis of the *Nnu*miR396a-GUS, *Nnu*miR396b-GUS, *NnSS2*-GUS and *NnPGM2*-GUS were performed [46,47]. *NtActin* was used as the reference gene. Three replicates were performed for each amplification reaction. We used SPSS software for statistical analysis of the qRT-PCR data.

### 4.8. Transient Dual-Luciferase Detection

To further confirm the binding activity of miRNA and target genes, dual luciferase assay was performed. The pre-*Nnu*miR396a/396b precursor were combined to pGreenII 62-SK-LUC (*Nnu*miR396a-LUC and *Nnu*miR396b-LUC) (Cat No.10911; Shanghai, China). The coding sequences of *NnSS2* and *NnPGM2* were combined into pGreenII 0800-LUC between (*NnSS2*-LUC and *NnPGM2*-LUC). The recombinant plasmids of miRNA and target genes were transferred into *Agrobacterium* strain GV3101. The bacteria were resuspended with the infection solution (100 mM Acetosyringone, 0.5 M MES (pH5.6) and 10 mM MgCl_2_) until the OD600 value was 1.0 and the bacterial solution containing miRNA and target genes were mixed in a ratio of 1:1, and then left to stand for 3 h. Five-week-old tobacco plants were infiltrated with the suspended bacterial solutions. According to the fluorescence value measured by the double reporting system, the fluorescence value of the target gene plasmid the fluorescence value of the internal reference plasmid (F/R value) was calculated, and the ratio of the target gene plasmid to the control group was calculated, the standard error was calculated with SPSS software and the histogram was made with GraphPad Prism 8.0 software. Total RNA extraction from tobacco leaves and real-time qRT-PCR analysis of the *Nnu*miR396a-LUC, *Nnu*miR396b-LUC, *NnSS2*-LUC and *NnPGM2*-LUC were performed [32,48]. Three replicates were performed for each amplification reaction. We used SPSS software for statistical analysis of the qRT-PCR data.

## 5. Conclusions

In our study, qRT-PCR analyses, transient co-expression experiment and dual luciferase assay showed a negative regulatory relationship between the two miRNAs and their target genes. Compared with the starch content of three internodes of rhizomes, we can further draw a conclusion that *Nnu*miR396a down regulated the expression of *NnSS2* and ultimately prevents the synthesis of amylopectin, and *Nnu*miR396b down regulated the expression of *NnPGM2* and ultimately prevents the synthesis of total starch. In summary, the regulatory network described here will be of great significance for further in-depth research into the molecular mechanism of starch biosynthesis in developing lotus rhizome. Therefore, a comprehensive study on the coordinated roles of maternal effect and starch biosynthetic pathways will be further considered for a better understanding of the mechanism of starch biosynthesis in lotus, which will facilitate the improvement of yield and quality in future lotus breeding programs.

## Data Availability

Not applicable.

## Supplementary Materials

The following supporting information can be downloaded at: https://www.mdpi.com/article/10.3390/ijms23147605/s1.
