# Supplementary figures and images for: Integrated mRNA and Small RNA Sequencing Reveals a microRNA Regulatory Network Associated with Starch Biosynthesis in Lotus (Nelumbo nucifera Gaertn.) Rhizomes
Source: Int J Mol Sci. 2022 Jul 9;23(14):7605. doi: 10.3390/ijms23147605 (PMC9318480; doi:10.3390/ijms23147605)

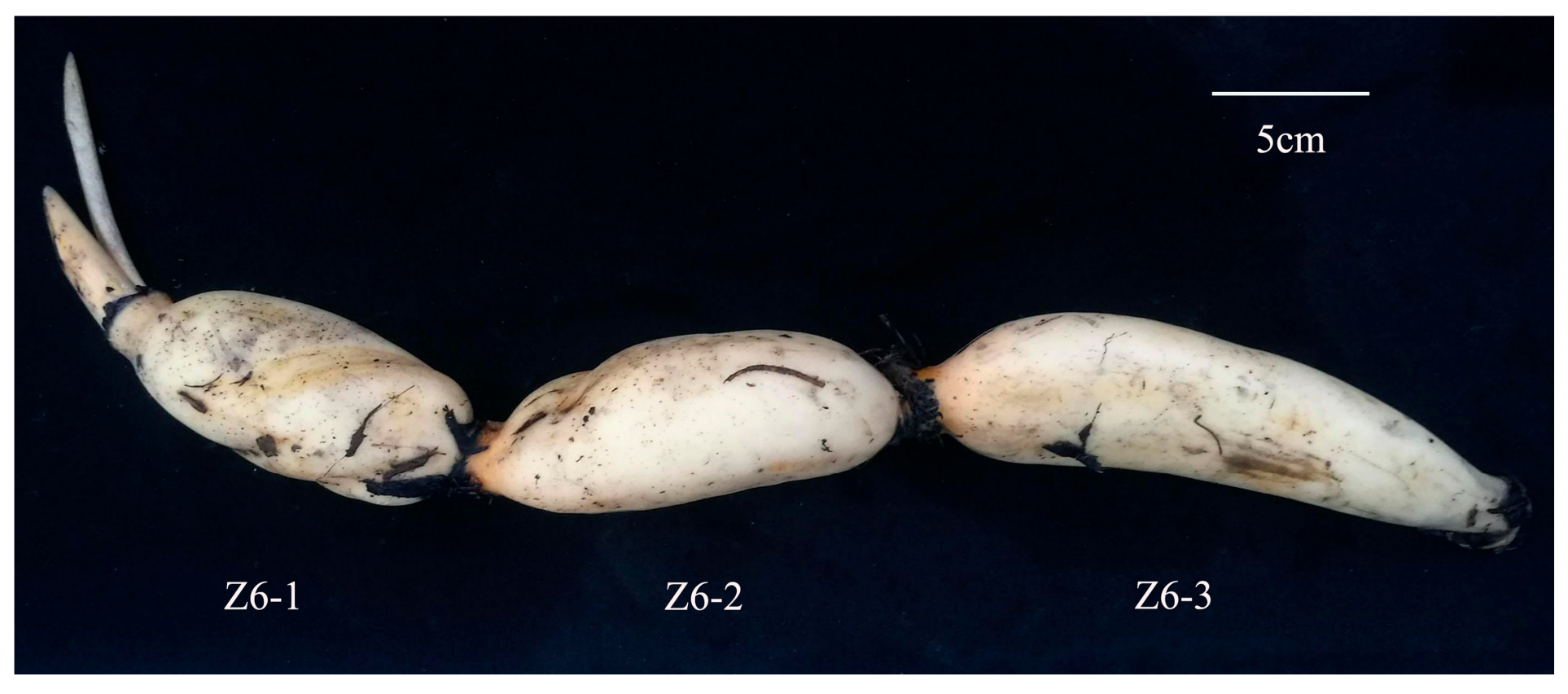

5cm

Z6-1

Z6-2

Z6-3

Supplement: Supplementary file 1 [file ijms-23-07605-s001.zip › Figure S1. Rhizome morphological of í░Z6í▒.pdf]
